# Effectiveness of multimodal participant recruitment in SPARK, a large, online longitudinal research study of autism

**DOI:** 10.1017/cts.2023.697

**Published:** 2023-12-14

**Authors:** Amy M. Daniels, J. Kiely Law, LeeAnne Green Snyder, Katharine Diehl, Robin P. Goin-Kochel, Pamela Feliciano, Wendy K. Chung, SPARK Consortium

**Affiliations:** 1 Simons Foundation, New York, NY, USA; 2 Kennedy Krieger Institute, Baltimore, MD, USA; 3 Department of Pediatrics, Baylor College of Medicine, Houston, TX, USA; 4 Department of Pediatrics, Boston Children’s Hospital, Harvard Medical School, Boston, MA, USA

**Keywords:** Recruitment, online research, social media, registry, pediatric, autism

## Abstract

**Background:**

SPARK launched in 2016 to build a US cohort of autistic individuals and their family members. Enrollment includes online consent to share data and optional consent to provide saliva for genomic analysis. SPARK’s recruitment strategies include social media and support of a nation-wide network of clinical sites. This study evaluates SPARK’s recruitment strategies to enroll a core study population.

**Methods:**

Individuals who joined between January 31, 2018, and May 29, 2019 were included in the analysis. Data include sociodemographic characteristics, clinical site referral, the website URL used to join, how the participant heard about SPARK, enrollment completion (online registration, study consents, and returning saliva sample), and completion of the baseline questionnaire. Logistic regressions were performed to evaluate the odds of core participant status (completing enrollment and baseline questionnaire) by recruitment strategy.

**Results:**

In total, 31,715 individuals joined during the study period, including 40% through a clinical site. Overall, 88% completed online registration, 46% returned saliva, and 38% were core participants. Those referred by a clinical site were almost twice as likely to be core participants. Those who directly visited the SPARK website or performed a Google search were more likely to be core participants than those who joined through social media.

**Discussion:**

Being a core participant may be associated with the “personal” connection and support provided by a clinical site and/or site staff, as well as greater motivation to seek research opportunities. Findings from this study underscore the value of adopting a multimodal recruitment approach that combines social media and a physical presence.

The SPARK study was launched in 2016 to recruit and retain a US cohort of autistic individuals and their family members [1]. Now with over 330,000 participants, including 130,000 autistic individuals, SPARK is the largest study of autism to date. As an online, recontactable cohort, SPARK represents a model for research infrastructure that enables researchers not only to access phenotypic and genetic data on thousands of individuals longitudinally but also to recruit individuals for additional research studies. As such, SPARK has become a catalyst for research and advancing the overall understanding of autism. For the research community more broadly, SPARK’s multimodal recruitment strategy can serve as a model for building other condition-specific, longitudinal research communities.

## The Evolution of Epidemiologic and Clinical Research in the USA

Research recruitment in the USA has evolved considerably as population demographics have shifted over time and with the advent of new technologies. Historically, participants may have been recruited in person, from targeted locations or through traditional outreach methods, such as mailings and telephone calls, both of which may limit sample size and participation from diverse groups of people and affect the overall generalizability of findings. Ongoing longitudinal studies have had to adapt. For instance, the Framingham Study has focused on the epidemiology of heart disease in several generations from a single community for over 70 years [2]. Over time, the study established two additional cohorts to address the racial and ethnic diversity limitations of the original cohort [3]. The Nurse’s Health Study is a longitudinal research study that began recruiting female nurses in the 1970s and has contributed significantly to knowledge of disease risk in women [4]. Now in its third phase, the study recruits nationally, includes both men and women, and is conducted entirely online, as compared to its original methodology that used a mailed survey [5].

## Large-Scale Adoption of Online Research and Recruitment

Outside of the aforementioned studies, the advent of the internet and the penetration of smartphones and social media have enabled the recruitment of large (100k+) cohorts and the efficient collection of a greater breadth of data (including genomic). Specifically, the use of web-based registries allows rapid collection of data on both common and rare diseases or conditions at scale [6]. Online research is not without its limitations, however, including biases in enrollment [7,8].

Online recruitment, particularly through digital advertising and social media, has grown significantly. Studies have found online recruitment methods to be more efficient and cost-effective in comparison to “offline” methods [9–12]. A review by Frampton and colleagues assessed the relative contribution of digital tools in both participant recruitment and retention in clinical trials [13]. Their review found that the use of digital tools doubled in the past decade (from 2008 to 2018), the most common being social media, internet sites, email, television/radio, and text messaging. Limitations include waning engagement over time [10], less representativeness (i.e., less racially/ethnically/linguistically diverse, and higher socioeconomic status [14,15]), and ineffectiveness in enrolling participants in clinical trials as compared to “offline” methods [9].

Of all social media channels, Facebook has been the most commonly utilized and effective recruitment platform [16,17]. Studies evaluating its effectiveness have found that paid ads using Facebook are superior in their ability to target a given geographic region or population [18–20], as well as re-engage participants who were lost to follow-up [17]. However, Facebook can be less cost-effective for recruiting diverse samples [19] or biased toward White, female participants [20].

## Recruitment of Vulnerable Populations

There are unique strategies and challenges associated with recruiting and retaining vulnerable populations in research. Regarding pediatric populations, the Healthy Communities Study [21] and National Children’s Study [22] are examples of epidemiological studies that recruited large, pediatric cohorts. Whereas the Healthy Communities Study recruited via schools, the National Children’s study adopted multipronged recruitment efforts that included household-based recruitment, provider-based recruitment, and direct outreach. Findings from these studies underscored the importance of adopting a multimodal approach to recruitment, particularly in obtaining a representative sample.

Challenges to online pediatric research include parent consent and pediatric assent [23,24], and in longitudinal studies, reconsenting and following children as they transition to adulthood. For instance, a pediatric biobank experienced challenges recruiting children, including re-consenting pediatric populations after they turned 18 [25]. Little is known about how best to recruit and retain emerging adults as well, but recent research suggests that recruitment through a range of strategies and engaging participants as partners may increase effectiveness [26].

Finally, a challenge that is not unique to pediatric research is the recruitment and engagement of traditionally underrepresented groups, such as individuals with disabilities and racial and ethnic minority populations [27]. Individuals with disabilities are routinely underrepresented in research because of physical, cognitive, and economic challenges and the added resources that may be required to accommodate their needs [28,29]. For racial and ethnic minority communities, studies have shown that it is important to employ a range of community engagement strategies [30,31], as well as communicate both their unique contributions to research and the benefits conferred with their participation [32–34].

## SPARK as a Model for Online, Longitudinal Research

Today there are several online, longitudinal studies collecting data (and in some cases, biosamples) on thousands of individuals. An example of a US-based study most comparable to SPARK in terms of recruitment methodology, size, and scope is the National Institutes of Health’s All of Us study [35]. The All of Us study aims to recruit one million individuals in the USA. Participants can join online or in-person at one of the partner clinical sites, and participation includes providing self-reported information online as well as biosamples. However, the study currently only recruits adults. An example of a condition-specific online registry that enrolls both children and adults is the T1D Exchange, for type I diabetes [36]. There are also many rare disease registries that focus on smaller, pediatric populations (e.g., Simons Searchlight [37], Angelman syndrome [38], and FORWARD for Fragile X [39]).

The SPARK study has parallels with the aforementioned studies insofar as it is online, longitudinal, and multifaceted in its collection of both self-report data and biospecimens and its ability to recontact individuals. However, SPARK is unique in adopting a multimodal approach to recruit children and adults with autism and their family members that includes both centralized recruitment through large-scale, digital media efforts and partnerships with over 30 clinical sites throughout the country. Herein, we describe the major recruitment strategies of SPARK and evaluate their relative effectiveness with respect to recruitment of a core study population.

## Materials and Methods

### Study Enrollment and Procedures

The SPARK study is funded by the Simons Foundation and uses a single, central IRB (WCG IRB Protocol #20151664). The study is open to all individuals with a professional diagnosis of autism and their family members who live in the USA and who read and understand English or Spanish. The qualifying, professional diagnosis of autism is based on self/proxy report at study entry.

**Figure 1.**
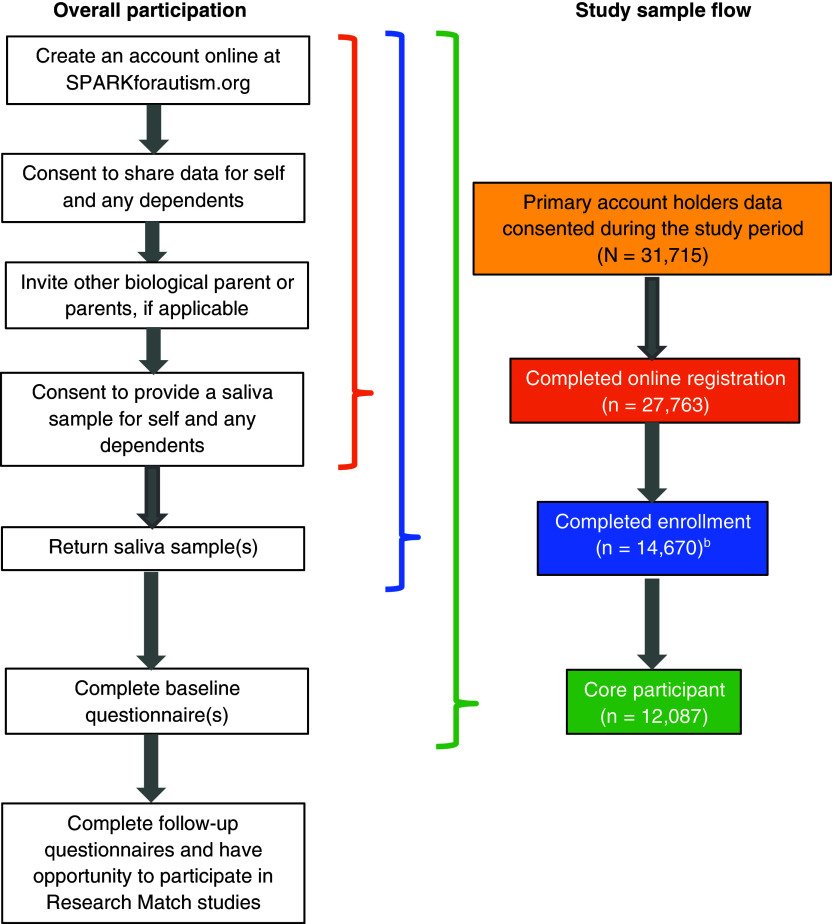
Overview of SPARK study participation for primary account holders^a^ and study sample flow. ^a^ The SPARK study participant who initiates enrollment in SPARK on behalf of themselves and their family members. ^b^ Not shown are 6,505 participants who are part of a “completed biological family,” whereby the primary account holder, secondary account holder, and individual with ASD have all completed enrollment.

An illustration of the major steps of SPARK study participation is presented in Figure 1. Parents/legal guardians of children and dependent adults with autism and independent autistic adults can enroll online at https://SPARKforAutism.org. After creating an account, the individual, herein referred to as the “primary account holder,” consents to share their data and to be recontacted about future research opportunities, and, if applicable, indicates their child/dependent’s assent to share information about themselves for research.

During registration, the “primary account holder” is also asked how they first heard about SPARK and is provided the following options: a clinical site/hospital/university, a community-based organization, the Interactive Autism Network (IAN), my healthcare provider (e.g., doctor or therapist), online (e.g., web page, Facebook, or other social media), through a media announcement (e.g., print, radio, or TV), a friend, invited by a family member, or other. The IAN, a similar online study, closed on June 30, 2019, and all existing participants were invited to join SPARK.

As a parent of a dependent child with autism, the “primary account holder” may also add non-autistic siblings of the individual with autism and is then asked to invite the other biological parent (or guardian), if available, to participate by providing their email address. The “primary account holder” must be over 18 years and, if a parent, the legally authorized representative of the child or dependent adult with autism. SPARK sends a separate email to the invited biological parent or “secondary account holder,” which includes instructions on how to join the study. The “primary account holder” and any minors/dependents are then invited to consent/assent to provide a saliva sample for DNA analysis and to receive genetic results, if desired. A saliva collection kit is shipped to the participant’s home at no cost to the family. Individuals are not required to participate in the genetic portion of the study to join SPARK. Autistic adult “primary account holders” follow a similar registration process whereby they consent for themselves and invite family members to participate.

Once online registration is complete, participants are asked to complete a series of demographic and behavioral or psychological questionnaires. The account dashboard is the participant’s study “home,” through which they access study consents, surveys, and tasks. Over time, participants may be invited to participate in additional research studies by external researchers through the SPARK Research Match program. Additional information about the SPARK study, including Research Match and the return of genetic findings to participants, can be found on the study’s website, https://sparkforautism.org/ [1].

### Recruitment Strategies

#### Clinical sites

SPARK funds a network of clinical recruitment sites throughout the USA. These sites are predominantly located at major academic medical centers that specialize in autism and other developmental disabilities. All sites have a site principal investigator and at least one research coordinator. Each clinical site has their own unique study URL (e.g., https://SPARKforAutism.org/TCH), which enables centralized tracking of all recruitment sites. The site’s primary role is to recruit individuals with autism and their biological family members into SPARK and support enrollment completion (e.g., assist with registration and/or saliva collection).

#### Digital advertising

SPARK advertises on Google, Bing, and through other platforms that utilize embedded algorithms to display ads for the SPARK study near similar (i.e., autism-focused) content. SPARK Google Ads Manager and Bing accounts display ads based on an autism-related search term or terms entered. Individuals also may learn about SPARK organically (i.e., through manual search).

#### Social media

SPARK has accounts on the following social media channels: Facebook, Instagram, Twitter, YouTube, and LinkedIn. Individuals can learn about and join SPARK organically (e.g., by viewing a friend’s post about SPARK in a feed) or by viewing and clicking a boosted post or paid ad on Facebook, Instagram, or YouTube. A boosted post differs from a paid ad in that it appears in SPARK’s newsfeed and can be delivered (or “boosted”) to a given audience for a fee. Ads have greater customization features but require setup through Meta’s Ads Manager program [40]. SPARK posts include static photos, GIFs, and videos and range in content from information about the SPARK study to person- or family-first accounts of their participation in SPARK.

#### Traditional and digital media

Since its inception, the SPARK study has been featured on national and local television, radio, and newspaper outlets, both print and digital. The SPARK central team typically drafts a press release, which is then added to an online press distribution platform and picked up by interested channels. SPARK has employed both marketing and public relations firms.

#### Organizational and community outreach

The major autism support and advocacy organizations in the USA, such as the Autism Society of America, as well as local, community-based groups, or individuals (i.e., bloggers) have links to SPARK included in their websites. Additionally, the following organizations have a unique study URL to enable tracking of SPARK participants through their specific channels: The Arc, Arkansas Autism Resource and Outreach Center, Autism Services & Resources Connecticut, Autism Speaks, Autism Society North Carolina, ASA Heartland, Easter Seals, GRASP, IAN, the Kentucky Autism Training Center, Mid-Michigan Autism Association, and Washington Autism Alliance & Advocacy.

### Measures

#### Participant characteristics

The study activities reported herein focused on the “primary account holder,” defined as the individual who first joins SPARK on behalf of the family and is assigned the majority of study tasks to complete on behalf of themselves and their dependents.

The following sociodemographic characteristics, collected during online registration or through subsequent study tasks, were used to characterize the “primary account holder:” age at registration; sex at birth; autism diagnosis (Y/N); ethnicity; race; US census region derived from participant-reported residence; metropolitan area based on 2013 Urban Influence Codes that define metropolitan counties by population size of their metro area [41]; and the area deprivation index (ADI). The ADI is derived from participant-reported addresses and constructed by ranking the ADI from low to high for the nation and grouping the block groups/neighborhoods into bins corresponding to each 1% range of the ADI. A block group with a ranking of 1 indicates the lowest level of “disadvantage” within the nation, and an ADI with a ranking of 100 indicates the highest level of “disadvantage” [42].

#### Recruitment strategies

We defined SPARK recruitment strategies in the following ways: (1) clinical site referral (Y/N); (2) the Hypertext Transfer Protocol (HTTP) referrer or the web address a user last visited before the SPARK site [43]; and (3) response to the single-choice question at the start of online registration, “How did you hear about us?” (see “Study enrollment and procedures” in Methods). Participants referred by a clinical site either clicked on or entered a site-specific URL in their browser or selected a specific clinical site from a dropdown menu. Free text responses from those who responded “other” to the question “How did you hear about us?” were then manually coded and grouped with either one of the aforementioned categories or labeled “unknown.” Available HTTP referrer links were manually grouped into the following mutually exclusive categories: Facebook or Instagram; Google or other search engine; SPARK website; clinical site URL; clinical site website; community organization; news story; invited parent link; and email link. The presence or absence of the HTTP referrer link (Y/N) was also coded. Missing URL information typically means that the origin site included code in the HTML that omits referrer information [43].

#### Enrollment

Enrollment completion was defined as a participant who completes online registration, including both the data and genetic consent, and returns their saliva kit.

#### Core study participant

As SPARK collects both phenotypic and genetic information from participants, the value of the data increases with the breadth and depth of information associated with each participant. Therefore, those who have provided a saliva sample in addition to completing a core set of tasks for SPARK are considered “core participants.” For this study, a core study participant is defined as the primary account holder who completes enrollment and the Basic Medical Screening Questionnaire (BMSQ; see supplemental materials). The BMSQ is available on the participant Dashboard immediately after completing registration, is administered to every SPARK participant, and includes questions about pregnancy, birth complications, medical issues, and developmental and behavioral conditions.

#### Complete family enrollment

As SPARK enables participation of the entire family, we also assessed complete family enrollment, defined as the fully consented, primary account holder, an invited second parent, and a child or dependent with autism who completed online registration and returned their saliva kits.

### Statistical Analysis

Descriptive analyses included measures of central tendency (e.g., means and proportions). Bivariate tests (e.g., chi-square and one-way analysis of variance tests) between the primary dependent variables (enrollment completion, core participant status, and family enrollment completion) and all participant characteristics were performed to identify which covariates to include in the multivariable regression analyses. Those with differences that were significant at a *p*-value of 0.05 or less were included in the multivariable models.

Multivariable logistic regression models were used to estimate the odds of enrollment completion, core participant status, and family enrollment by recruitment strategy. For these models, clinical site referral, the website used to join SPARK, and how a participant heard about SPARK were used as distinct primary independent variables. If a participant joined through a clinical site URL, they were automatically assigned “clinical site/hospital/university” in the “How did you hear about us?” dropdown menu, irrespective of whether they may have heard about SPARK in other ways. In contrast, for those *not* referred by a clinical site, participants were able to select from any of the options presented. Therefore, the relationships between how a participant heard about SPARK and the outcome measures were examined *only* in those who joined from the community at large (i.e., not referred by a clinical site).

For this study, we focused on core participant status as the primary outcome of interest, reporting only key differences observed from the regression models using enrollment completion and complete family enrollment. Further, in order to examine how the relationships between our recruitment strategies and primary outcome of interest differed in primary account holders with and without a self-reported autism diagnosis, stratified analyses were also performed, and only key differences are reported herein. Detailed findings related to enrollment completion and complete family enrollment for the entire sample and related to core participant status for autistic and non-autistic account holders are presented in supplemental tables.

Lastly, during the period analyzed herein, race and ethnicity information was only collected via a Dashboard questionnaire called the “Background History Questionnaire.” Because of the relatively low completion rate for that questionnaire, race and ethnicity data were missing on roughly 74% of participants. While these variables were included in the analysis to better understand the relationship between race and ethnicity and our primary outcome measures, we appreciate that this variable is also a confounder, as providing the information in and of itself may be considered a proxy for increased study engagement. Therefore, for each relationship examined, we presented findings from two multivariable regression models – one with race and ethnicity and one without. SPARK data release version 9 was used and analyzed with Stata/SE version 18.0 [44].

### Sample

As of July 2023, there were a total of 189,000 account holders in SPARK (excluding all dependents, i.e., minors with and without autism). While the study has been recruiting participants since December 2015, it started large-scale digital and social media advertising in February 2018. In addition, on May 29, 2019, every individual who joined SPARK was automatically referred to a clinical site based on their zip code. Prior to this change, participants were linked to a clinical site only if they joined through a unique site URL or selected “clinical site/hospital/university” from the “How did you hear about us?” question. A total of 64,762 individuals created an account during the period analyzed herein.

In order to evaluate the associations with joining through a clinical site or other method, the study sample was restricted to all primary account holders, that is, the independent adult who first joined SPARK on behalf of his or her family members and responsible for completing the majority of study tasks, who joined after January 31, 2018 and before May 29, 2019. The final study sample included 31,715 data consented, primary account holders. Lastly, the sample did not include account holders who were recruited into SPARK but subsequently chose to withdraw or whose data were held back from public release due to identified phenotypic data flags (2,065 as of July 2023).

## Results

### Participant Characteristics

The average age at study registration for the primary account holder was 38.5 years (SD 9.0), and 86% were female (Table 1). Eight percent self-reported an autism diagnosis. Among the 26% who reported ethnicity and race, 15% were Hispanic and the majority were White (80%). The plurality of participants reported living in the South (37%), with only 12% in a non-Metropolitan area. The mean ADI was 48.8 (SD 25.7), indicating slightly lower deprivation compared to the median of 50.0.

**Table 1.** Characteristics of primary account holders^a^ in SPARK (*N* = 31,715)

Characteristic		
Age at registration, years, mean (SD)	38.5	(9.0)
Sex at birth, *N* (%)		
Male	4295	(14%)
Female	27,420	(86%)
Autism spectrum disorder diagnosis, *N* (%)		
No	29,326	(92%)
Yes	2,389	(8%)
Race and/or ethnicity reported, *N* (%)		
No	23,434	(74%)
Yes	8,281	(26%)
Race, *N* (%), *n* = 8,242		
White only	6,570	(80%)
African American only	493	(6%)
Asian only	275	(3%)
Native American/Native Hawaiian only	73	(1%)
Other	439	(5%)
More than one race	392	(5%)
Hispanic ethnicity, *N* (%), *n* = 8,281		
No	7,053	(85%)
Yes	1,228	(15%)
US census region, *N* (%), *n* = 28,426		
Northeast	4,795	(17%)
Midwest	7,083	(25%)
South	10,600	(37%)
West	5,948	(21%)
Metropolitan area, *N* (%), *n* = 31,598		
No	3,767	(12%)
Yes	27,831	(88%)
Area deprivation index national rank, percent, mean (SD), *n* = 24,606	48.8	(25.7)

a The SPARK study participant who initiates enrollment in SPARK on behalf of themselves and their family members.

### Study Completion

Of the 31,715 primary account holders who joined the SPARK study during the study period, 88% completed online registration (and both the data and genetic consents), 46% completed enrollment (online registration and returned saliva sample), and 38% were defined as core participants (Fig. 1). Lastly, 21% of all primary account holders were part of complete families (completed enrollment for both biological parents and the child with autism).

### Recruitment Strategies

**Figure 2.**
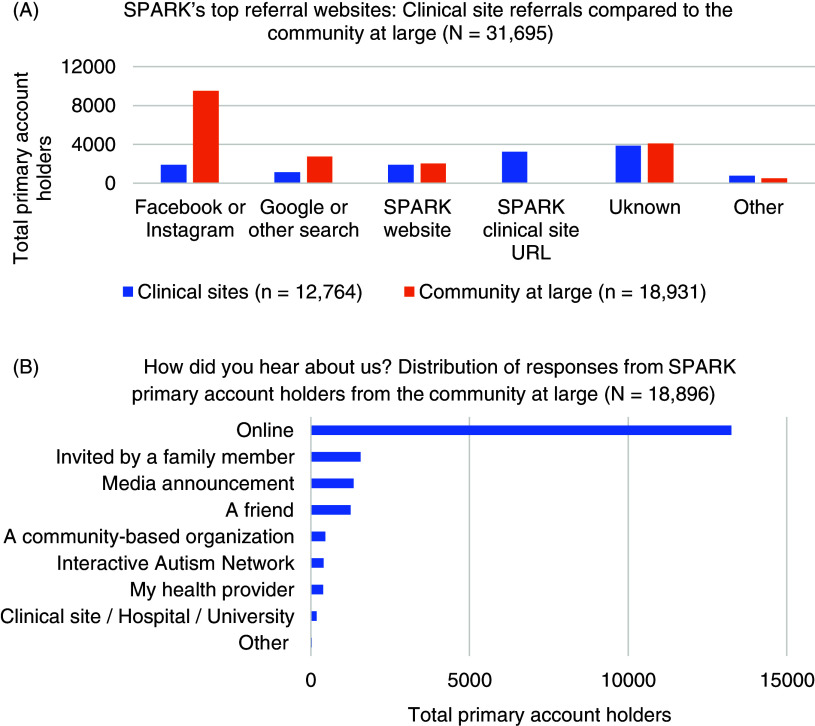
Recruitment sources in SPARK^a^. ^a^ Recruitment sources for primary account holders, defined as the SPARK study participant who initiates enrollment in SPARK on behalf of themselves and their family members, include **(A)** the referral website used by SPARK participants who joined through a clinical site versus the community at large (*n* = 31,695; missing data excluded) and **(B)** the response to “How did you hear about us?” from the community at large only (*n* = 18,896; unknown responses are not included). Individuals who joined SPARK through a clinical site were automatically assigned to the “clinical site/hospital/university” response category and are therefore not represented here.

With respect to recruitment method (Fig. 2), 40% of all participants were referred by clinical site. Of participants with available URL data (75%), the top websites used to join the study were Facebook or Instagram (48%), the SPARK website (16%), Google or other search engines (16%), and SPARK clinical site URLs (14%). When including those whose URL data were unknown, the top three reported referral sites were Facebook and Instagram (36%), Unknown (25%), and the SPARK website and Google and other search (both at 12%). Among participants who joined from the community at large, most heard about SPARK online (70%), followed by being invited by a family member (8%), or through a media announcement (7%).

### Clinical Site Referral and Core Participant Status

**Figure 3.**
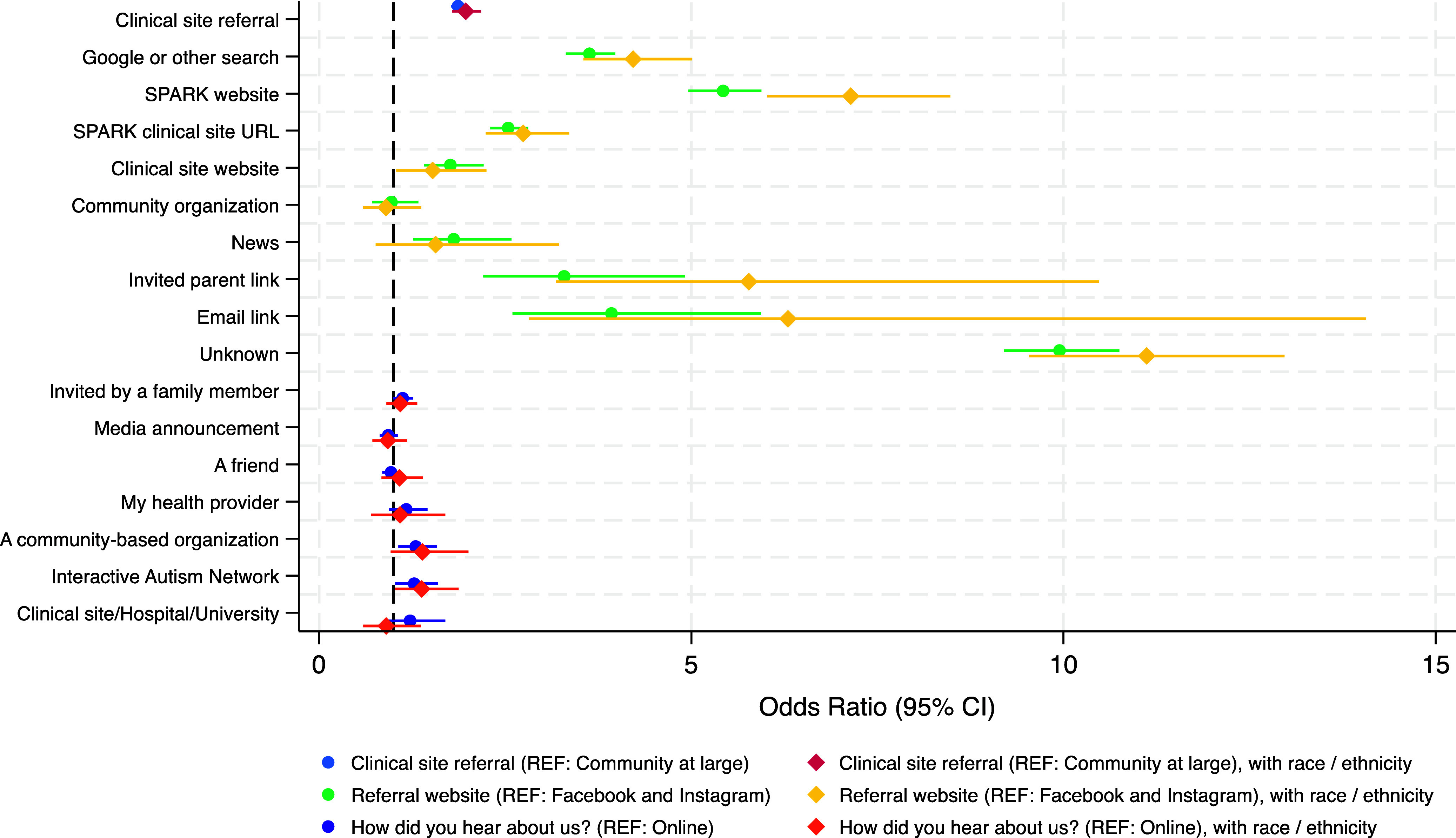
Adjusted odds of core participant status among primary account holders in SPARK, by recruitment method (*N* = 31,715)^a^. ^a^ The SPARK study participant who initiates enrollment in SPARK on behalf of themselves and their family members; How did you hear about us? Include the community at large only (*N* = 18,945); CI = confidence interval; REF = reference group; all models adjusted for sex at birth, age at registration, autism spectrum disorder diagnosis, area deprivation index national rank, and US census region.

In both models with and without race and ethnicity, clinical site referral was associated with a two times increased odds of being a core participant, adjusting for autism diagnosis, age at registration, census region, ADI, and in model 2, race and ethnicity (Table 2; Fig. 3). For both models, an autism diagnosis was associated with an increased odds of being a core participant, as was living in the Midwest or West, as compared to the East. In model 2, both African American race and Hispanic ethnicity were associated with a significant decreased odds of core participant status.

**Table 2.** The relationship between recruitment method and core participant status among primary account holders^a^ in SPARK (N = 31,715)

	Clinical site referral OR (95% CI)		Referral website OR (95% CI)		How did you hear about us?^b^ OR (95% CI)	
	Model 1^c^	Model 2^d^	Model 1^c^	Model 2^d^	Model 1^c^	Model 2^d^
**Clinical site referral**	1.9 (1.8, 2.0)**	2.0 (1.8, 2.2)**	–	–	–	–
**Referral site**						
Facebook or Instagram	–	–	1.0	1.0	–	–
Google or other search	–	–	3.6 (3.3, 4.0)**	4.2 (3.5, 5.0)**	–	–
SPARK website	–	–	5.4 (4.9, 5.9)**	7.0 (5.9, 8.3)**	–	–
SPARK clinical site URL	–	–	2.5 (2.2, 2.8) **	2.6 (2.2, 3.2)**	–	–
Clinical site website	–	–	1.8 (1.4, 2.2) **	1.5 (1.0, 2.2)*	–	–
Community organization	–	–	1.0 (0.7, 1.3)	0.9 (0.6, 1.4)	–	–
News	–	–	1.8 (1.3, 2.6) **	1.6 (0.8, 3.2)	–	–
Invited parent link	–	–	3.0 (2.0, 4.4) **	5.2 (2.9, .9.3)**	–	–
Email link	–	–	3.9 (2.6, 5.9) **	6.3 (2.8, 14.0)**	–	–
Unknown	–	–	9.9 (9.2, 10.7)**	11.0 (9.4, 12.8)**	–	–
**How did you hear about us?**						
Online					1.0	1.0
Invited by family member	–	–	–	–	1.1 (1.0, 1.3)	1.1 (0.9, 1.3)
Media announcement	–	–	–	–	0.9 (0.8, 1.1)	0.9 (0.7, 1.2)
A friend	–	–	–	–	1.0 (0.9, 1.1)	1.1 (0.8, 1.4)
My health provider	–	–	–	–	1.2 (0.9, 1.5)	1.1 (0.7, 1.7)
Community-based organization	–	–	–	–	1.3 (1.1, 1.6)**	1.4 (1.0, 2.0)
Interactive Autism Network	–	–	–	–	1.3 (1.0, 1.6)*	1.4 (1.0, 1.9)*
Clinical site/hospital/university	–	–	–	–	1.2 (0.9, 1.7)	0.9 (0.6, 1.4)
**Covariates**						
***Age at registration, years***	1.0 (1.0, 1.0)**	1.0 (1.0, 1.0)	1.0 (1.0, 1.0)**	1.0 (1.0, 1.0)	1.0 (1.0, 1.0)*	1.0 (1.0, 1.0)*
***Autism spectrum disorder diagnosis***	2.0 (1.8, 2.2)**	3.8 (3.3, 4.3)**	1.7 (1.5, 1.9)**	3.1 (2.7, 3.6)**	2.2 (2.0, 2.5)**	4.2 (3.6, 4.9)**
***US census region***						
East	1.0	1.0	1.0	1.0	1.0	1.0
Midwest	1.3 (1.2, 1.4)**	1.2 (1.1, 1.4)**	1.3 (1.2, 1.4)**	1.3 (1.1, 1.6)**	1.3 (1.1, 1.4)**	1.2 (1.0, 1.5)
South	1.1 (1.0, 1.1)	1.1 (1.0, 1.3)	1.1 (1.0, 1.2)*	1.1 (1.0, 1.3)	1.1 (1.0, 1.2)*	1.1 (0.9, 1.3)
West	1.2 (1.1, 1.3)**	1.3 (1.1, 1.5)**	1.2 (1.1, 1.3)*	1.3 (1.1, 1.6)**	1.2 (1.0, 1.3)**	1.3 (1.0, 1.5)*
***Area deprivation index national rank percent***	1.0 (1.0, 1.0)	1.0 (1.0, 1.0)	1.0 (1.0, 1.0)**	1.0 (1.0, 1.0)	–	–
***Race***						
White only	–	1.0	–	1.0	–	1.0
African American only	–	0.6 (0.5, 0.8)**	–	0.6 (0.5, 0.8)**	–	0.7 (0.5, 0.9)*
Asian only	–	0.8 (0.6, 1.0)	–	0.7 (0.5, 1.0)*	–	0.7 (0.5, 1.2)
Native American/Hawaiian only	–	0.8 (0.5, 1.5)	–	0.8 (0.5, 1.5)	–	1.0 (0.6, 2.0)
Other	–	0.9 (0.7, 1.2)	–	0.9 (0.7, 1.2)	–	0.8 (0.6, 1.1)
More than one race	–	1.0 (0.8, 1.3)	–	1.0 (0.8, 1.3)	–	0.9 (0.7, 1.2)
***Hispanic ethnicity***	–	0.7 (0.6, 0.8)**	–	0.7 (0.6, 0.8)**	–	0.8 (0.6, 1.0)**
** *p* < 0.01 * *p* < 0.05						

a The SPARK study participant who initiates enrollment in SPARK on behalf of themselves and their family members.

b Community at large only (*N* = 18,945).

c Without race and ethnicity.

d With race and ethnicity.

### Referral Site and Core Participant Status

Compared to joining through Facebook or Instagram, participants were significantly more likely to be core participants if they joined through Google, the SPARK website, a SPARK clinical site URL, an invited parent link, or an email link in both models (Table 2; Fig. 3). Joining from a news story was associated with a significant increased odds of enrollment completion in model 1 only. For both models, an autism diagnosis was associated with an increased odds of being a core participant, as was living in the Midwest or West. In model 2, African American race, Asian race, and Hispanic ethnicity were all associated with a significant decreased odds of being a core participant.

### How a Participant Heard about SPARK and Core Participant Status Among the Community at Large

Compared to hearing about SPARK “online,” the only sources significantly associated with an increased odds of core participant status were the IAN and community-based organizations (Table 2; model 1 only; Fig. 3). In both models, a self-reported autism diagnosis and living in the West (Midwest and South in model 1 only) were associated with an increased odds of core participant status. African American race and Hispanic ethnicity were associated with a decreased odds of being a core participant in model 2.

### Key Differences Using Enrollment Completion and Complete Family Enrollment as Outcomes

The relationships between clinical site referral and enrollment completion (Supplementary Table s1) and clinical site referral and complete family enrollment (Supplementary Table s2) were stronger compared to the observed site referral and core participant relationship. An autism diagnosis, older age at registration, and male sex at birth were all associated with a decreased odds of family enrollment, whereas Asian race was associated with an increased odds. In the referral site and family enrollment models, the same relationships between the aforementioned covariates were observed. Lastly, in the how a participant heard about us and family enrollment model, Asian race was not associated with complete family enrollment.

### Recruitment Strategies and Core Participant Status Stratified by Autism Diagnosis

The relationship between clinical site referral and core participant status among non-autistic primary account holders (Supplementary Table s3) was comparable to that observed in the combined analysis and moderately attenuated in the autistic only sample (Supplementary Table s4). In the referral site and core participant model, directly visiting the SPARK website, being invited by another parent, and clicking on an email link (vs. social media) were the strongest predictors of core participant status for the non-autistic samples. For autistic primary account holders, Google or other search, directly visiting the SPARK website and using an invited parent link (model 2 only) were the strongest predictors of core participant status. For the non-autistic primary account holders who were not referred by a clinical site (i.e., from the community at large), hearing about SPARK through a community-based organization (model 1) or IAN (model 2) were associated with an increased odds of core participant status. For the autistic adult account holders, IAN was the only predictor of core participant status (model 1).

## Discussion

Overall, primary account holders (parents of a dependent with autism or an independent adult with autism) who completed online registration, provided a biospecimen, and completed the baseline questionnaire, defined as “core participants” in this study, were more likely to have been referred by clinical site and clicked on a link other than Facebook or Instagram. These same participants were also significantly more likely to live in the Midwest or Western regions of the USA and less likely to be African American and Hispanic. Among those coming from the community at large rather than from a clinical site, both community-based organizations and a referral from the IAN were associated with increased likelihood of reaching core participant status.

Findings from this study suggest that having personal assistance from or some connection to a clinical site enhances study enrollment and task completion in online research. In particular, complex, multistep enrollment processes and family member enrollment may be more readily completed with the support of in-person study staff to facilitate participant completion of study tasks. Furthermore, despite high fixed personnel costs, the effectiveness of in-person recruitment may be worthwhile if large numbers of participants can be enrolled at the site.

There may also be greater trust among potential participants to join and remain engaged in a study if it is associated with a known medical institution or their own healthcare provider. The same logic may also apply to participants who heard about SPARK from the IAN. Those recruited at large who first heard about SPARK through IAN were significantly more likely to be core participants, particularly autistic adults. While their study engagement in SPARK may be confounded by their previous participation in autism research, referral by a trusted source, and not necessarily in-person, may be an important factor for some groups, particularly the autistic adult community.

Overall, while this study demonstrated that participants referred by clinical sites were more likely to complete enrollment, be core participants, and complete family enrollment, the exact strategies employed by the SPARK clinical sites were not assessed here. However, more detailed analysis of recruitment strategies used by SPARK clinical sites and how both research staff and participants perceived these approaches were assessed in our companion paper (unpublished data); results corroborate our current findings that personal support offered by research teams, particularly in connection with participants’ medical providers, can successfully engage and retain participants through study completion. Additional research is needed to better understand the different approaches that clinical sites undertook to recruit these participants.

The value of digital media, and social media in particular, to participant recruitment in online research should not be understated. The overwhelming majority of participants heard about SPARK “online,” and over 35% joined through Facebook or Instagram. Other studies have found that recruitment through social media channels like Facebook and Instagram are efficient [10,12] and result in the largest pool of eligible participants compared to other methods [11,45]. As demonstrated in this study, however, social media alone does not result in a greater likelihood of enrollment or study task completion as compared to online searchers for the SPARK study or visiting the study site directly.

Meta-analyses support that adopting multiple recruitment strategies, such as combinations of “online” and “offline” or “active” (i.e., direct outreach) and “passive” (i.e., digital or out of home advertisements) methods, increase the likelihood of meeting recruitment goals [9,46]. Whether a study chooses to adopt recruitment strategies that are resource-intensive, such as employing in-person study personnel, or those that are more scalable and reach larger numbers, such as social media advertising, will depend largely on the goals of the study and burden of study participation in the short and long term. Additionally, there are other factors, including time, geography, and characteristics of a given study population that will likely influence which recruitment strategies to adopt. In a study like SPARK that enables a participant to, in essence, “choose your own adventure,” we found that a multimodal approach to recruitment was needed. Findings from this study demonstrate that a mix of both high- and low-resource-intensive strategies is optimal for recruiting large numbers of participants who are requested to complete multiple study tasks, including providing a biospecimen.

With respect to the characteristics of SPARK account holders who were more likely to become core participants, findings are consistent with other studies that show that engaged participants, particularly those recruited online and/or participating in online research, are more likely to be female and White [7,14,20]. In our study, African American and Hispanic participants were significantly less likely to complete enrollment, reach core participant status, or complete family enrollment. Interestingly, while Asian families in SPARK were less likely to reach core participant status, they were more likely to have complete family enrollment. These different outcomes speak to a need to develop specific, culturally informed approaches to recruitment and engagement in research for distinct communities versus a “one-size-fits-all” approach. Indeed, in recent years, there has been more research on effective engagement of racial and ethnic minority communities that highlight the need for more localized, participatory, and community-informed strategies to recruit and retain under-represented groups [34,47]. In an effort to increase representation of these communities and build a cohort that more closely resembles the US population, SPARK has recently implemented a comprehensive diversity, equity, and inclusivity (DEI) initiative that includes additional support to clinical sites, targeted marketing campaigns, and a DEI advisory board. Research is needed to evaluate the effectiveness of these efforts in recruiting a representative cohort. Ultimately, studies like SPARK have a responsibility to work closely with key stakeholders and community groups, not only to overcome structural barriers to study participation, like access to the internet, but also to address the nuanced cultural barriers and historical trauma experienced by so many communities, to achieve true representation in research.

When compared to findings from other contemporaneous, national online disease registries, many of SPARK’s findings are comparable. For instance, the American Cancer Society’s (ACS) Cancer Prevention Study 3, which enrolled over 300,000 individuals at highly publicized and well-staffed ACS events throughout the country, found few differences comparing participants who partially versus fully enrolled [48]. However, like the SPARK study, they observed significantly greater participation among White females. The Sister Study Cohort recruited over 50,000 females across the USA using several diverse recruitment methods [49]. Like the SPARK and CPS-3 cohorts, participants were more likely to be non-Hispanic, White, and similar to SPARK to be recruited from the Midwest and Southern regions of the USA. In the Brain health registry of over 100,000 participants, African American, Asian, and Latino participants were significantly less likely than their White counterparts to complete the baseline questionnaires, comparable to this study’s findings related to core participants [50]. Furthermore, the overwhelming majority of participants were female. Similar to the aforementioned studies, the Alzheimer’s Prevention Registry, a study of over 300,000 individuals, was comprised of predominantly White, non-Hispanic females [51]. Like SPARK, the APR employed a number of different recruitment strategies, of which paid social was responsible for bringing in the plurality (39%) of participants. Nonetheless, a significant proportion of those who joined through social media failed to reengage over time. Collectively, like SPARK, these studies succeeded in their efforts to recruit tens of thousands of individuals by employing a range of both national and geographically targeted passive and active recruitments studies. However, they all observed disparities in participation by gender, race, and ethnicity, which in many cases, extended to outcomes related to study task completion and longitudinal engagement. A comprehensive assessment of what these and other studies, particularly those with a focus on reaching underrepresented groups, have implemented and evaluated in this area may help to inform future efforts at achieving greater representation in disease registry research.

Findings from this study should be interpreted in the context of several limitations. First, the SPARK cohort is based on parent- or self-reported autism diagnosis, and diagnoses have not been systematically validated across the entire cohort. However, a recent verification study using electronic medical record data was able to confirm autism in 98.8% of a SPARK sample [52]. Second, SPARK is not a population-based study and, as such, findings are not representative of the entire population of individuals with autism and their families in the USA. However, characteristics of children with autism in the SPARK sample, such as the ratio of males to females, and age at diagnosis, closely mirror those of other large cohorts (i.e., the CDC Autism and Developmental Disabilities Monitoring Network [53]). Lastly, an important consideration in identifying and prioritizing recruitment strategies is cost, which this study did not assess.

Since its national launch in 2016, the SPARK study has enrolled hundreds of thousands of research participants and their family members by employing a multifaceted recruitment strategy that combines a national network of clinical sites with large-scale digital and social media outreach. SPARK’s multimodal recruitment strategy can serve as a model for building other complex, longitudinal research communities.

## Supporting information

Daniels et al. supplementary material Daniels et al. supplementary material

## Supplementary material

The supplementary material for this article can be found at https://doi.org/10.1017/cts.2023.697.
