# Supplementary material for: Effectiveness of multimodal participant recruitment in SPARK, a large, online longitudinal research study of autism
Source: J Clin Transl Sci. 2023 Dec 14;8(1):e64. doi: 10.1017/cts.2023.697 (PMC11036434; doi:10.1017/cts.2023.697)
Supplement: Daniels et al. supplementary material [file S2059866123006970sup001.docx]

| **Table S1.** The relationship between recruitment method and enrollment completion among primary account holders^a^ in SPARK (N = 31,715) | | | | | | |  |  |  |  |  |
| --- | --- | --- | --- | --- | --- | --- | --- | --- | --- | --- | --- |
|  | **Clinical site referral**  **OR (95% CI)** | | **Referral website**  **OR (95% CI)** | | **How did you hear about us?**^b^  **OR (95% CI)** | | |  |  |  |  |
|  | Model 1^c^ | Model 2^d^ | Model 1^c^ | Model 2^d^ | Model 1^c^ | Model 2^d^ | |  |  |  |  |
| **Clinical site referral** | 2.9 (2.8, 3.1)** | 3.1 (2.8, 3.5)** | - | - | - | - | |  |  |  |  |
| **Referral site** |  |  |  |  |  |  | |  |  |  |  |
| Facebook or Instagram |  |  | 1.0 | 1.0 |  |  | |  |  |  |  |
| Google or other search | - | - | 3.4 (3.1, 3.7) ** | 3.9 (3.3, 4.6)** | - | - | |  |  |  |  |
| SPARK website | - | - | 6.3 (5.8, 6.9)** | 8.2 (6.9, 9.7)** | - | - | |  |  |  |  |
| SPARK clinical site URL | - | - | 6.1 (5.5, 6.8)** | 6.5 (5.3, 7.9)** | - | - | |  |  |  |  |
| Clinical site website | - | - | 1.9 (1.5, 2.3)** | 1.7 (1.2, 2.5)** | - | - | |  |  |  |  |
| Community organization | - | - | 0.9 (0.7, 1.2) | 0.9 (0.6, 1.3) | - | - | |  |  |  |  |
| News | - | - | 1.8 (1.3, 2.5)** | 1.4 (0.7, 2.8) | - | - | |  |  |  |  |
| Invited parent link | - | - | 3.9 (2.6, 5.8)** | 6.7 (3.6, 12.5)** | - | - | |  |  |  |  |
| Email link | - | - | 3.8 (2.5, 5.8)** | 6.2 (2.7, 14.2)** | - | - | |  |  |  |  |
| Unknown | - | - | 10.2 (9.4, 11.0)** | 11.4 (9.8, 13.2)** | - | - | |  |  |  |  |
| **How did you hear about us?** |  |  |  |  |  |  | |  |  |  |  |
| Online | - | - | - | - | 1.0 | 1.0 | |  |  |  |  |
| Invited by family member | - | - | - | - | 1.1 (1.0, 1.3) | 1.1 (0.9, 1.4) | |  |  |  |  |
| Media announcement | - | - | - | - | 1.0 (0.9, 1.1) | 1.0 (0.8, 1.3) | |  |  |  |  |
| A friend | - | - | - | - | 1.0 (0.8, 1.1) | 1.1 (0.9, 1.4) | |  |  |  |  |
| My health provider | - | - | - | - | 1.3 (1.0, 1.6)* | 1.3 (0.8, 1.9) | |  |  |  |  |
| Community-based organization | - | - | - | - | 1.2 (1.0, 1.5)* | 1.5 (1.0, 2.2)* | |  |  |  |  |
| Interactive Autism Network | - | - | - | - | 1.2 (1.0, 1.5) | 1.3 (1.0, 1.8) | |  |  |  |  |
| Clinical site / Hosp. / University | - | - | - | - | 1.2 (0.9, 1.7) | 1.0 (0.6, 1.5) | |  |  |  |  |
| **Covariates** |  |  |  |  |  |  | |  |  |  |  |
| ***Age at registration, years*** | - | - | - | - | 1.0 (1.0, 1.0)** | 1.0 (1.0, 1.0)** | |  |  |  |  |
| ***Male sex at birth*** | 0.9 (0.8, 0.9)** | 1.0 (0.8, 1.1) | 0.9 (0.9, 1.0) | 1.0 (0.9, 1.2) | 1.0 (0.9, 1.1) | 1.1 (0.9, 1.3) | |  |  |  |  |
| ***Autism spectrum disorder diagnosis*** | 2.1 (1.9, 2.3)** | 3.3 (2.9, 3.8)** | 1.8 (1.6, 2.0)** | 2.6 (2.2, 3.1)** | 2.2 (2.0, 2.5)** | 3.8 (3.2, 4.5)** | |  |  |  |  |
| ***United States census region*** |  |  |  |  |  |  | |  |  |  |  |
| East | 1.0 | 1.0 | 1.0 | 1.0 | 1.0 | 1.0 | |  |  |  |  |
| Midwest | 1.3 (1.2, 1.4)** | 1.2 (1.0, 1.4)* | 1.3 (1.2, 1.4)** | 1.3 (1.1, 1.5)** | 1.3 (1.1, 1.4)** | 1.3 (1.0, 1.5)* | |  |  |  |  |
| South | 1.0 (1.0, 1.1) | 1.0 (0.8, 1.1) | 1.1 (1.0, 1.1) | 1.0 (0.8, 1.1) | 1.1 (1.0, 1.3)* | 1.1 (1.0, 1.4) | |  |  |  |  |
| West | 1.1 (1.0, 1.2) | 1.0 (0.9, 1.2) | 1.1 (1.0, 1.2)* | 1.1 (0.9, 1.3) | 1.1 (1.0, 1.3)* | 1.3 (1.0, 1.6)* | |  |  |  |  |
| ***Area Deprivation Index national rank percent*** | 1.0 (1.0, 1.0)** | 1.0 (1.0, 1.0)** | 1.0 (1.0, 1.0)** | 1.0 (1.0, 1.0)** | 1.0 (1.0, 1.0)* | 1.0 (1.0, 1.0)* | |  |  |  |  |
| ***Race*** |  |  |  |  |  |  | |  |  |  |  |
| White only | - | 1.0 | - | 1.0 | - | 1.0 | |  |  |  |  |
| African American only | - | 0.8 (0.6, 1.0)* | - | 0.8 (0.7, 1.0) | - | 0.8 (0.6, 1.1) | |  |  |  |  |
| Asian only | - | 1.3 (1.0, 1.8) | - | 1.3 (0.9, 1.7) | - | 1.1 (0.6, 1.7) | |  |  |  |  |
| Native American/Hawaiian only | - | 0.7 (0.4, 1.2) | - | 0.7 (0.4, 1.2) | - | 0.8 (0.4, 1.6) | |  |  |  |  |
| Other | - | 1.2 (1.0, 1.6) | - | 1.2 (0.9, 1.5) | - | 1.0 (0.7, 1.4) | |  |  |  |  |
| More than one race | - | 1.1 (0.9, 1.4) | - | 1.1 (0.9, 1.4) | - | 0.9 (0.7, 1.2) | |  |  |  |  |
| ***Hispanic ethnicity*** | - | 0.8 (0.7, 0.9)** | - | 0.8 (0.7, 1.0)* | - | 0.8 (0.6, 1.0)* | |  |  |  |  |
| ** *p*<.01 * *p*<.05 | | | | | | | | |  |  | ** p<.01 * p<.05 |

^a^ The SPARK study participant who initiates enrollment in SPARK on behalf of themselves and their family members; ^b^ Community at large only (N = 18,945); ^c^ Without race and ethnicity; ^d^ With race and ethnicity

| **Table S2.** The relationship between recruitment method and complete family enrollment among primary account holders^a^ in SPARK (N = 31,715) | | | | | | |
| --- | --- | --- | --- | --- | --- | --- |
|  | **Clinical site referral**  **OR (95% CI)** | | **Referral website**  **OR (95% CI)** | | **How did you hear about us?**^b^  **OR (95% CI)** | |
|  | Model 1^c^ | Model 2^d^ | Model 1^c^ | Model 2^d^ | Model 1^c^ | Model 2^d^ |
| **Clinical site referral** | 3.8 (3.5, 4.0)** | 4.3 (3.8, 4.8)** | - | - | - | - |
| **Referral site** |  |  |  |  |  |  |
| Facebook or Instagram | - | - | 1.0 | 1.0 | - | - |
| Google or other search | - | - | 3.7 (3.3, 4.2)** | 4.4 (3.4, 5.6)** | - | - |
| SPARK website | - | - | 6.4 (5.7, 7.2)** | 8.0 (6.3, 10.2)** | - | - |
| SPARK clinical site URL | - | - | 7.4 (6.6, 8.4)** | 8.2 (6.3, 10.5)** | - | - |
| Clinical site website | - | - | 3.5 (2.7, 4.6)** | 4.3 (2.7, 6.7)** | - | - |
| Community organization | - | - | 0.9 (0.6, 1.5) | 1.0 (0.5, 1.9) | - | - |
| News | - | - | 3.5 (2.3, 5.3)** | 2.7 (1.1, 6.5)** | - | - |
| Invited parent link | - | - | 6.8 (4.4, 10.6)** | 7.1 (4.0, 13.8)** | - | - |
| Email link | - | - | 4.8 (3.0, 7.7)** | 7.9 (3.3, 18.9)** | - | - |
| Unknown | - | - | 7.6 (6.9, 8.5)** | 8.8 (7.0, 10.9)** | - | - |
| **How did you hear about us?** |  |  |  |  |  |  |
| Online | - | - | - | - | 1.0 | 1.0 |
| Invited by family member | - | - | - | - | 1.2 (1.0, 1.4) | 1.4 (1.1, 1.9)** |
| Media announcement | - | - | - | - | 1.0 (0.8, 1.2) | 1.2 (0.9, 1.8) |
| A friend | - | - | - | - | 1.1 (0.9, 1.3) | 1.2 (0.9, 1.8) |
| My health provider | - | - | - | - | 1.5 (1.1, 2.0)** | 1.3 (0.7, 2.4) |
| Community-based organization | - | - | - | - | 1.6 (1.2, 2.1)** | 2.1 (1.3, 3.4)** |
| Interactive Autism Network | - | - | - | - | 0.9 (0.6, 1.3) | 1.2 (0.8, 2.0) |
| Clinical site / Hosp. / University | - | - | - | - | 1.8 (1.2, 2.7)** | 1.7 (1.0, 2.9)** |
| **Covariates** |  |  |  |  |  |  |
| ***Age at registration, years*** | - | - | 1.0 (1.0, 1.0) | 1.0 (1.0, 1.0) | 1.0 (1.0, 1.0)** | 1.0 (1.0, 1.0)* |
| ***Sex at birth*** | 0.6 (0.5, 0.6)** | 0.6 (0.5, 0.7)** | 0.6 (0.6, 0.7)** | 0.6 (0.5, 0.7)** | 0.7 (0.6, 0.8)** | 0.6 (0.5, 0.9)** |
| ***Autism spectrum disorder diagnosis*** | 0.3 (0.3, 0.4)** | 0.3 (0.2, 0.4)** | 0.2 (0.2, 0.3)** | 0.2 (0.2, 0.3)** | 0.4 (0.3, 0.5)** | 0.4 (0.3, 0.6)** |
| ***Metropolitan area*** | 1.0 (1.0, 1.0) | 1.0 (1.0, 1.0) | 1.0 (0.9, 1.1) | 1.2 (0.9, 1.4) | - | - |
| ***United States census region*** |  |  |  |  |  |  |
| East | 1.0 | 1.0 | 1.0 | 1.0 | 1.0 | 1.0 |
| Midwest | 1.2 (1.1, 1.3)** | 1.2 (1.0, 1.4) | 1.2 (1.1, 1.4)** | 1.3 (1.1, 1.5)** | 1.3 (1.1, 1.6)** | 1.2 (0.9, 1.6) |
| South | 1.1 (1.0, 1.1) | 0.9 (0.7, 1.0) | 1.0 (0.9, 1.2) | 0.8 (0.7, 1.0)* | 1.1 (1.0, 1.3) | 0.9 (0.6, 1.2) |
| West | 1.1 (1.0, 1.2) | 0.9 (0.8, 1.1) | 1.1 (1.0, 1.2) | 1.0 (0.8, 1.2) | 1.1 (0.9, 1.3) | 1.1 (0.8, 1.5) |
| ***Area Deprivation Index national rank percent*** | 1.0 (1.0, 1.0)** | 1.0 (1.0, 1.0)** | 1.0 (1.0, 1.0)** | 1.0 (1.0, 1.0)** | 1.0 (1.0, 1.0)** | 1.0 (1.0, 1.0)** |
| ***Race*** |  |  |  |  |  |  |
| White only | - | 1.0 | - | 1.0 | - | 1.0 |
| African American only | - | 0.5 (0.4, 0.6)** | - | 0.5 (0.4, 0.7)** | - | 0.4 (0.2, 0.7)** |
| Asian only | - | 1.4 (1.1, 1.9)** | - | 1.5 (1.1, 2.0)** | - | 1.0 (0.5, 1.8) |
| Native American/Hawaiian only | - | 0.5 (0.2, 1.3) | - | 0.5 (0.2, 1.3) | - | 0.6 (0.2, 2.0) |
| Other | - | 0.8 (0.6, 1.1) | - | 0.8 (0.6, 1.1) | - | 0.6 (0.4, 1.0) |
| More than one race | - | 0.9 (0.7, 1.2) | - | 0.9 (0.6, 1.2) | - | 0.9 (0.6, 1.4) |
| ** *p*<.01 * *p*<.05 | | | | | | |

^a^ The SPARK study participant who initiates enrollment in SPARK on behalf of themselves and their family members; ^b^ Community at large only (N = 18,945); ^c^ Without race and ethnicity; ^d^ With race; ethnicity was not significant in the bivariate analyses

| **Table S3.** The relationship between recruitment method and core participant status among non-autistic primary account holders^a^ in SPARK (N = 29,326) | | | | | | |
| --- | --- | --- | --- | --- | --- | --- |
|  | **Clinical site affiliation**  **OR (95% CI)** | | **Referral website**  **OR (95% CI)** | | **How did you hear about us?**^b^  **OR (95% CI)** | |
|  | Model 1^c^ | Model 2^d^ | Model 1^c^ | Model 2^d^ | Model 1^c^ | Model 2^d^ |
| **Clinical site referral** | 1.9 (1.8, 2.0)** | 2.0 (1.8, 2.2)** | - | - | - | - |
| **Referral site** |  |  |  |  |  |  |
| Facebook or Instagram | - | - | 1.0 | 1.0 | - | - |
| Google or other search | - | - | 3.6 (3.3, 4.0)** | 4.3 (3.6, 5.2)** | - | - |
| SPARK website | - | - | 5.2 (4.7, 5.7)** | 6.7 (5.5, 8.1)** | - | - |
| SPARK clinical site URL | - | - | 2.6 (2.3, 2.8) ** | 2.8 (2.2, 3.4)** | - | - |
| Clinical site website | - | - | 1.8 (1.4, 2.3)** | 1.4 (0.9, 2.3) | - | - |
| Community organization | - | - | 1.0 (0.8, 1.4) | 1.0 (0.7, 1.6) | - | - |
| News | - | - | 1.9 (1.3, 2.7)** | 1.5 (0.7, 3.3) | - | - |
| Invited parent link | - | - | 4.4 (2.8, 7.2) ** | 6.7 (3.4, .13.4)** | - | - |
| Email link | - | - | 4.2 (2.7, 6.6) ** | 8.6 (3.5, 21.4)** | - | - |
| Unknown | - | - | 9.7 (8.9, 10.5)** | 11.0 (9.4, 13.1)** | - | - |
| **How did you hear about us?** |  |  |  |  |  |  |
| Online |  |  |  |  | 1.0 | 1.0 |
| Invited by family member | - | - | - | - | 1.1 (1.0, 1.3) | 1.1 (0.9, 1.4) |
| Media announcement | - | - | - | - | 0.9 (0.8, 1.1) | 0.9 (0.7, 1.2) |
| A friend | - | - | - | - | 1.0 (0.9, 1.1) | 1.1 (0.8, 1.5) |
| My health provider | - | - | - | - | 1.2 (0.9, 1.5) | 1.1 (0.7, 1.9) |
| Community-based organization | - | - | - | - | 1.3 (1.1, 1.6)* | 1.3 (0.8, 1.9) |
| Interactive Autism Network | - | - | - | - | 1.2 (1.0, 1.5) | 1.4 (1.0, 2.0) |
| Clinical site / Hosp. / University | - | - | - | - | 1.3 (0.9, 1.8) | 1.0 (0.6, 1.6) |
| **Covariates** |  |  |  |  |  |  |
| ***Age at registration, years*** | 1.0 (1.0, 1.0)** | 1.0 (1.0, 1.0)* | 1.0 (1.0, 1.0)** | 1.0 (1.0, 1.0)* | 1.0 (1.0, 1.0) | 1.0 (1.0, 1.0)** |
| ***United States census region*** |  |  |  |  |  |  |
| East | 1.0 | 1.0 | 1.0 | 1.0 | 1.0 | 1.0 |
| Midwest | 1.3 (1.2, 1.4)** | 1.2 (1.1, 1.5)** | 1.3 (1.2, 1.4)** | 1.4 (1.1, 1.7)** | 1.3 (1.1, 1.4)** | 1.3 (1.0, 1.6) |
| South | 1.1 (1.0, 1.1) | 1.1 (0.9, 1.3) | 1.1 (1.0, 1.2) | 1.1 (1.0, 1.3) | 1.1 (1.0, 1.2) | 1.0 (0.8, 1.3) |
| West | 1.1 (1.0, 1.2)* | 1.3 (1.1, 1.5)** | 1.2 (1.1, 1.3)** | 1.4 (1.1, 1.6)** | 1.1 (1.0, 1.3) | 1.2 (1.0, 1.6) |
| ***Area Deprivation Index national rank percent*** | 1.0 (1.0, 1.0) | 1.0 (1.0, 1.0) | 1.0 (1.0, 1.0)* | 1.0 (1.0, 1.0) | - | - |
| ***Race*** |  |  |  |  |  |  |
| White only | - | 1.0 | - | 1.0 | - | 1.0 |
| African American only | - | 0.6 (0.5, 0.7)** | - | 0.6 (0.4, 0.7)** | - | 0.7 (0.5, 0.9)* |
| Asian only | - | 0.8 (0.6, 1.0)* | - | 0.7 (0.5, 0.9)* | - | 0.6 (0.4, 1.1) |
| Native American/Hawaiian only | - | 0.9 (0.5, 1.9) | - | 1.0 (0.5, 2.1) | - | 1.1 (0.5, 2.4) |
| Other | - | 1.0 (0.8, 1.3) | - | 0.9 (0.7, 1.2) | - | 0.9 (0.6, 1.3) |
| More than one race | - | 1.1 (0.9, 1.4) | - | 1.0 (0.8, 1.4) | - | 1.0 (0.7, 1.3) |
| ***Hispanic ethnicity*** | - | 0.7 (0.6, 0.8)** | - | 0.7 (0.6, 0.8)** | - | 0.8 (0.6, 1.0)** |
| ** *p*<.01 * *p*<.05 | | | | | | |

^a^ The SPARK study participant who initiates enrollment in SPARK on behalf of themselves and their family members; ^b^ Community at large only (N = 17,359);^c^ Without race and ethnicity; ^d^ With race and ethnicity­­­­

| **Table S4.** The relationship between recruitment method and core participant status among autistic primary account holders^a^ in SPARK (N = 2,389) | | | | | | |
| --- | --- | --- | --- | --- | --- | --- |
|  | **Clinical site referral**  **OR (95% CI)** | | **Referral website**  **OR (95% CI)** | | **How did you hear about us?**^b^  **OR (95% CI)** | |
|  | Model 1^c^ | Model 2^d^ | Model 1^c^ | Model 2^d^ | Model 1^c^ | Model 2^d^ |
| **Clinical site referral** | 1.4 (1.2, 1.7)** | 1.7 (1.3, 2.2)** | - | - | - | - |
| **Referral site** |  |  |  |  |  |  |
| Facebook or Instagram | - | - | 1.0 | 1.0 | - | - |
| Google or other search | - | - | 3.8 (2.8, 5.3)** | 3.6 (2.4, 5.4)** | - | - |
| SPARK website | - | - | 7.9 (5.8, 10.8)** | 8.3 (5.6, 12.2)** | - | - |
| SPARK clinical site URL | - | - | 1.7 (1.1, 2.7) * | 1.7 (1.0, 3.0)* | - | - |
| Clinical site website | - | - | 1.7 (0.8, 3.4) | 1.5 (0.7, 3.4) | - | - |
| Community organization | - | - | 0.6 (0.2, 1.8) | 0.5 (0.2, 1.3) | - | - |
| News | - | - | 1.5 (0.4, 5.9) | 2.3 (0.4, 13.9) | - | - |
| Invited parent link | - | - | 1.4 (0.7, 3.0) | 2.7 (0.9, 7.7) | - | - |
| Email link | - | - | 1.9 (0.5, 6.9) | 2.3 (0.5, 10.6) | - | - |
| Unknown | - | - | 13.9 (10.1, 19.1)** | 10.4 (7.1, 15.2)** | - | - |
| **How did you hear about us?** |  |  |  |  |  |  |
| Online |  |  |  |  | 1.0 | 1.0 |
| Invited by family member | - | - | - | - | 1.1 (0.8, 1.4) | 1.0 (0.7, 1.4) |
| Media announcement | - | - | - | - | 0.7 (0.5, 1.1) | 0.9 (0.5, 1.7) |
| A friend | - | - | - | - | 0.9 (0.6, 1.4) | 0.9 (0.5, 1.6) |
| My health provider | - | - | - | - | 1.1 (0.4, 2.6) | 0.9 (0.3, 2.4) |
| Community-based organization | - | - | - | - | 2.0 (1.0, 4.3) | 2.0 (0.7, 5.3) |
| Interactive Autism Network | - | - | - | - | 2.0 (1.0, 3.4)* | 1.2 (0.6, 2.5) |
| Clinical site / Hosp. / University | - | - | - | - | 0.8 (0.3, 2.1) | 0.4 (0.1, 1.0) |
| **Covariates** |  |  |  |  |  |  |
| ***Age at registration, years*** | 1.0 (1.0, 1.0) | 1.0 (1.0, 1.0) | 1.0 (1.0, 1.0) | 1.0 (1.0, 1.0) | 1.0 (1.0, 1.0) | 1.0 (1.0, 1.0) |
| ***United States census region*** |  |  |  |  |  |  |
| East | 1.0 | 1.0 | 1.0 | 1.0 | 1.0 | 1.0 |
| Midwest | 1.0 (0.8, 1.4) | 1.1 (0.8, 1.6) | 1.2 (0.9, 1.7) | 1.2 (0.8 1.9) | 1.1 (0.8, 1.5) | 1.1 (0.7, 1.7) |
| South | 1.1 (0.9 1.5) | 1.2 (0.9, 1.7) | 1.2 (0.9, 1.6) | 1.3 (0.9 1.9) | 1.3 (1.0, 1.9) | 1.3 (0.9, 2.0) |
| West | 1.3 (1.0, 1.7) | 1.3 (0.9, 1.8) | 1.4 (1.0, 1.9) | 1.3 (0.8, 1.9) | 1.6 (1.1, 2.2)* | 1.4 (0.9, 2.2) |
| ***Area Deprivation Index national rank percent*** | 1.0 (1.0, 1.0) | 1.0 (1.0, 1.0) | 1.0 (1.0, 1.0) | 1.0 (1.0, 1.0) | - | - |
| ***Race*** |  |  |  |  |  |  |
| White only | - | 1.0 | - | 1.0 | - | 1.0 |
| African American only | - | 1.2 (0.6, 2.5) | - | 1.1 (0.5, 2.4) | - | 1.0 (0.4, 2.4) |
| Asian only | - | 1.4 (0.4, 5.1) | - | 1.4 (0.4, 5.3) | - | 3.6 (0.4, 30.1) |
| Native American/Hawaiian only | - | 0.7 (0.3, 1.8) | - | 0.6 (0.2, 1.5) | - | 1.1 (0.4, 2.9) |
| Other | - | 0.7 (0.4, 1.3) | - | 0.8 (0.4, 1.6) | - | 0.6 (0.3, 1.3) |
| More than one race | - | 0.8 (0.5, 1.2) | - | 0.9 (0.5, 1.5) | - | 0.7 (0.4, 1.2) |
| ***Hispanic ethnicity*** | - | 0.6 (0.4, 0.9)** | - | 0.6 (0.4, 1.0) | - | 0.9 (0.5, 1.4) |
| ** *p*<.01 * *p*<.05 | | | | | | |

^a^ The SPARK study participant who initiates enrollment in SPARK on behalf of themselves and their family members; ^b^ Community at large only (N = 1,586);^c^ Without race and ethnicity; ^d^ With race and ethnicity­­­­
